# Effect of cue-based feeding on time to nipple feed and time to discharge in very low birth weight infants

**DOI:** 10.1038/s41598-023-36634-y

**Published:** 2023-06-12

**Authors:** Jonathan Spagnoli, Ramasubbareddy Dhanireddy, Emily Gannon, Sandeep Chilakala

**Affiliations:** 1 Department of Pediatrics, Division of Neonatology, The University of Tennessee Health Science Center, 201 Rout Center for Women and Newborns, 853 Jefferson Avenue, Memphis, TN 38103 USA; 2 Regional One Health Rout Center for Women and Newborns, Memphis, TN USA

**Keywords:** Paediatrics, Neonatology

## Abstract

The objective of this study is to evaluate the effectiveness of a cue-based feeding protocol in improving time to nipple feed and time to discharge in very low birth weight infants in a Level III Neonatal Intensive Care Unit. Demographic, feeding, and discharge data were recorded and compared between the two cohorts. The pre-protocol cohort included infants born from August 2013 through April 2016 and the post-protocol cohort included infants born from January 2017 through December 2019. 272 infants were included in the pre-protocol cohort and 314 infants in the post-protocol cohort. Both cohorts were statistically comparable in gestational age, gender, race, birthweight, prenatal care, antenatal steroid use, and rates of maternal diabetes. There were statistically significant differences between the pre- versus post-protocol cohorts in median post-menstrual age (PMA) in days at first nipple feed (PO) (240 vs 238, *p* = 0.025), PMA in days at full PO (250 vs 247, *p* = 0.015), and length of stay in days (55 vs 48, *p* = 0.0113). Comparing each year in the post-protocol cohort, for each outcome measure, a similar trend was noted in 2017 and 2018, but not in 2019. In conclusion, the cue-based feeding protocol was associated with a decrease in the time to first PO, time to full nipple feeds, and the length of stay in very-low-birthweight infants.

## Introduction

In 2008, the American Academy of Pediatrics (AAP) stated that for medical discharge readiness, a potential high-risk Neonatal Intensive Care Unit (NICU) graduate needs to display temperature homeostasis, maturity of respiratory control, consistent pattern of appropriate weight gain, stability in supine sleeping position, and maturity of oral feeding skills^1^. The suck–swallow–breath pattern is not fully developed in an infant until around 32–34 weeks post-menstrual age (PMA)^2^. While seemingly simple for a healthy individual, this coordinated action can be difficult for preterm infants, due to differences in anatomy and neural physiology^3–5^. Attempts to nipple feed when not fully developed can lead to fatigue, risk of aspiration, and bradycardia during feeding^6,7^. This in turn can lead to increased length of stay and increased risk of readmission post NICU discharge^8^. Traditional methods of gauging the readiness of an infant for PO is based primarily on weight, gestational age, and providers' assessment of readiness. These approaches depend on physician orders, providers' experience, and intuitional guidelines^9^. Furthermore, the gauge of a successful nipple feeding was based on adequate volume intake at scheduled times, regardless of infant behavior or provider manipulation^10^. PO feeding in preterm infants with preset volumes when developmentally immature leads to increased stress and delayed time to successful PO^11^. Transitioning gavage feeds to PO based on developmental cues is called cue-based feeding. Previously, evidence showed that feeding based on behavioral cues could be an effective way to gauge the readiness of a preterm infant for PO^2,10^. Cue-based feeding, which allows an infant to PO when developmentally ready, is based on a study by Ludwig et al.^10^ that outlines the scale and its use in practice. According to the study, a successful feed in an infant-driven approach must be safe, functional, nurturing, and individually/developmentally appropriate. Their scale has 3 sections: feeding readiness scale, quality of nippling scale, and caregiver technique scale. Feeding readiness and quality of nippling scales are ordinal values with 1 being optimal and 5 being the least optimal. The caregiver technique scale has nominal values that describe various actions the caregiver could complete to assist in the feed. The goal of this method was a more infant development-cue-driven and objective approach to determine and document the readiness of an infant to nipple feed.

Several NICUs started implementing cue-based feeding transition and studies were published regarding its effectiveness. Some earlier studies showed a reduction in time to PO and length of stay after implementing the cue-based protocol^12,13^. Evidence about cue-based feeding effectiveness is conflicting, as the studies included infants across various gestational ages, mostly moderately preterm and late preterm, who don’t represent the most at-risk patients, and the feeding outcomes assessed are not uniform across the studies^14–16^.

A variety of approaches are used in hospitals to initiate cue-based feeding, and our unit adopted the one from the Ludwig et al. study^10^. This study aims to assess whether implementation of a cue-based feeding protocol is associated with reduced time to first PO feeds, full PO feeds, and length of NICU stay in very low birth weight (VLBW) infants at Regional One Health.

## Methods

This study was approved by the Institutional Review Board at the University of Tennessee Health Science Center through an exempt process. Additionally, informed consent was waived by the Institutional Review Board at the University of Tennessee Health Science Center, as this is a retrospective study based on deidentified data from the Regional One Health patient database. All methods were performed in accordance with the relevant guidelines and regulations.

This is a retrospective study including VLBW infants born at the Regional One Health NICU between August 2013 until December 2019. Infants with congenital anomalies, necrotizing enterocolitis, severe intraventricular hemorrhage (grade III and grade IV), and any other anomaly that could affect feeding were excluded. Infants were divided into two cohorts. The pre-protocol cohort (before implementing the cue-based feeding protocol) included infants born from August 2013 through April 2016. The post-protocol cohort (after implementing the cue-based feeding protocol) included infants born from January 2017 through December 2019. The primary objective of the study was to determine if change of practice to a cue-based feeding protocol was associated with improved time to first PO, time to full PO, and decreased length of stay in the NICU.

Data on variables of interest including gestational age (GA), birth weight (BW), gender, race, prenatal care, antenatal steroids, and maternal diabetes were collected. In addition, data on neonatal feeding outcome variables including the date at the first readiness score, date at first PO feeds, date at full PO feeds, and length of stay in the NICU were collected. Demographic and outcomes data were compared between the two cohorts.

The chi-squared test was used for nominal data, the Mann–Whitney test for continuous data between 2 groups, and the Kruskal–Wallis test for continuous data between more than two groups. The Man–Whitney and Kruskal–Wallis tests were used due to the skewed nature of the data. We considered a *p* value of less than 0.05 to be statistically significant. Data analysis involved the use of Graph Pad Prism (GraphPad Software ® San Diego, California, USA).

### Ethics approval and consent to participate

This study was approved by the Institutional Review Board at the University of Tennessee Health Science Center through an exempt process. Informed consent was waived by the Institutional Review Board at the University of Tennessee Health Science Center, as this was a retrospective study based on deidentified patient data.

## Results

**Figure 1:**
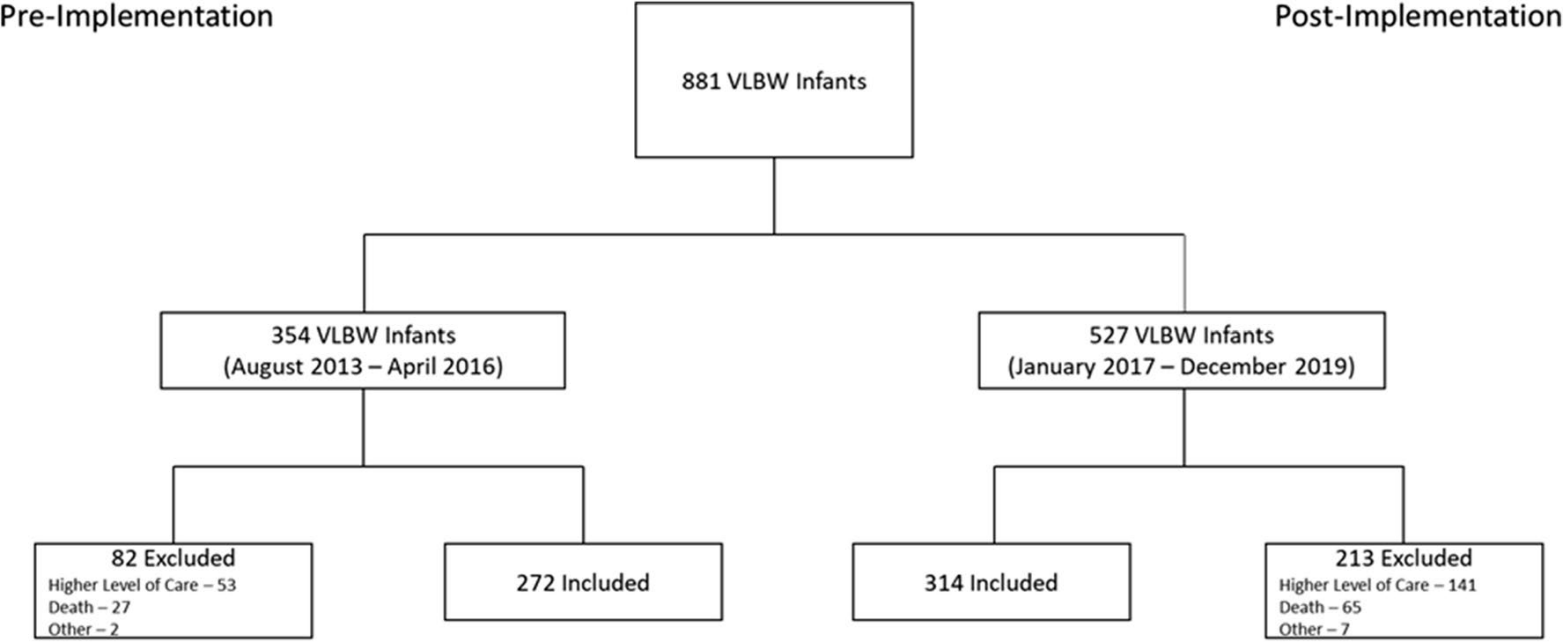
Infants enrolled in the study. This diagram displays how the final participants in the study were included based on inclusion and exclusion criteria.

A total of 354 infants in the pre-protocol cohort and 527 in the post-protocol cohort were identified. After exclusions, 272 in the pre-protocol cohort and 314 in the post-protocol cohort were included in the study (Fig. 1). The median GA was 29.2 (IQR: 27.3–31.1) in the pre- and 29.6 (IQR: 28.0–31.4) in the post-protocol cohorts. The median BW was 1150 g (880–1358) in the pre- and 1180 g (897.5–1370) in the post-protocol cohorts. Both cohorts were statistically comparable in gestational age, birthweight, gender, race, prenatal care, antenatal corticosteroid use, and rates of maternal diabetes (Table 1).

**Table 1:** Clinical characteristics of the study cohorts.

	Pre-protocol (n = 272)	Post-protocol (n = 314)	*p* value
GA (weeks. days), median (IQR)	29.25 (27.3–31.18)	29.6 (28.0–31.4)	0.06
Birthweight (g), median (IQR)	1150 (880–1358)	1180 (898–1370)	0.36
Male—N%	52.9%	46.8%	0.13
Maternal race (African American)—N%	86.4%	85%	0.63
Prenatal care—N%	93.%	92.6%	0.87
Antenatal steroids—N%	91.1%	93.6%	0.26
Maternal diabetes—N%	9.9%	9.2%	0.77

Demographics of both cohorts. This data shows there were no statistically significant differences in basic demographics between the two groups.

**Figure 2:**
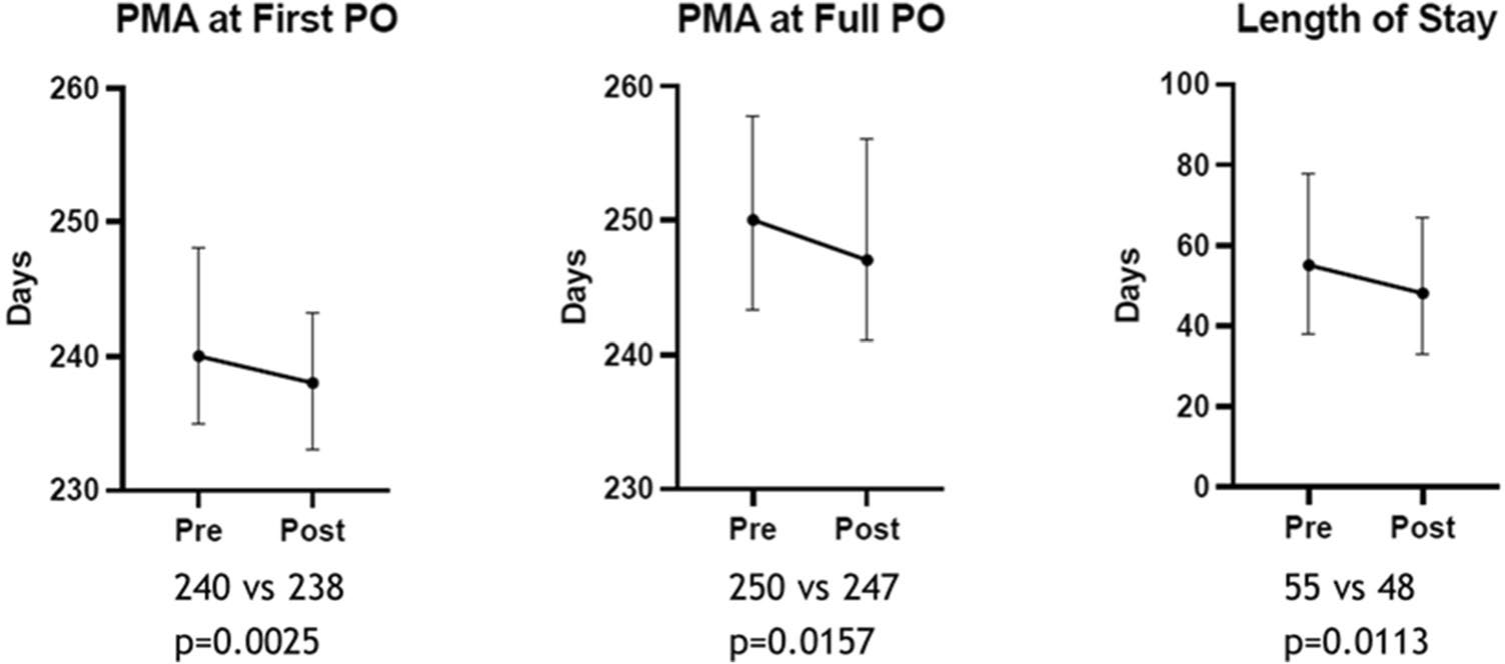
Outcomes of the study cohorts. These graphs compare the pre- (N = 272) and post-cue-based feeding (N = 314) cohorts on feeding and length of stay outcomes. The values of PMA are in days. Medians are shown, and the error bars are the interquartile ranges.

The PMA in days at first PO in the pre-protocol cohort, 240 (IQR:235–248), had a statistically significant difference from post-protocol cohort, 238 (IQR:233–243.3), *p* = 0.0025. Similarly, statistically significant differences between the two cohorts were noted in median PMA in days at full PO feeds, 250 (IQR:243–257) vs 247 (IQR: 241–256), *p* = 0.0157, and median length of stay in days, 55 (IQR:38–77.75) vs 48 days (IQR: 33–67), *p* = 0.0113 (Fig. 2).

**Figure 3:**
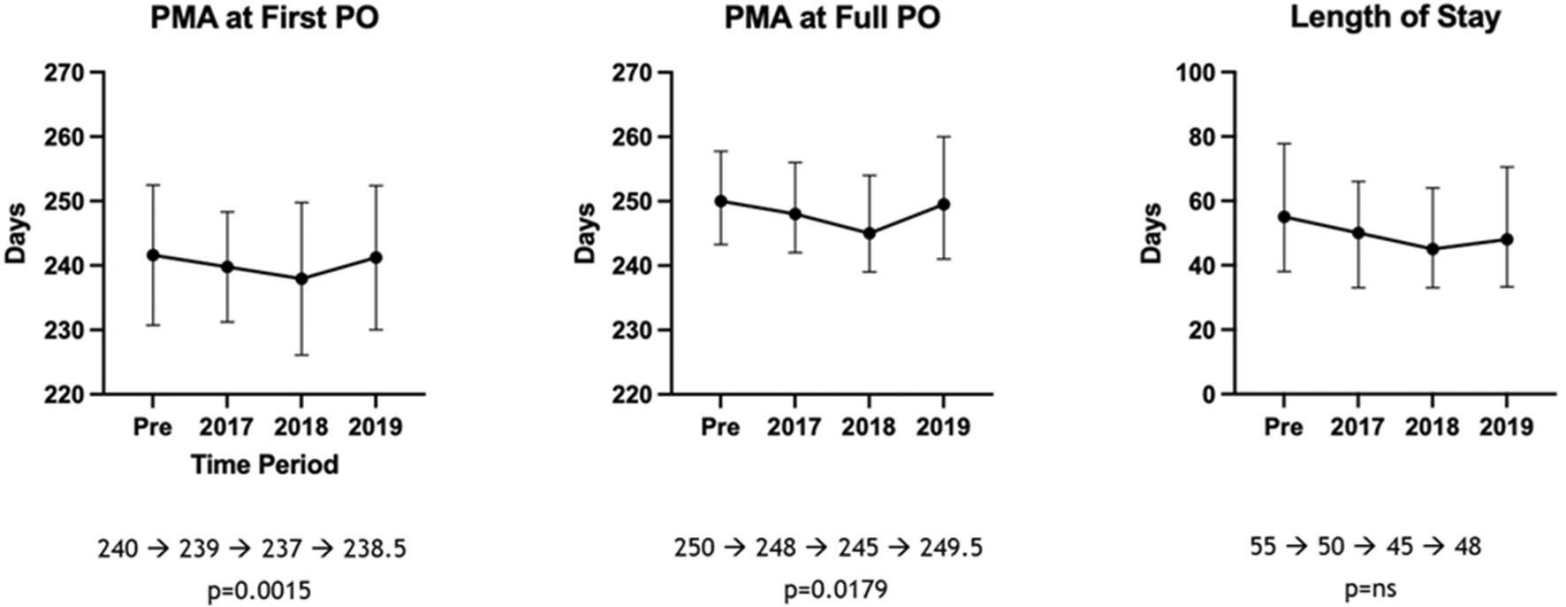
Outcomes of the post implementation cohort by each year. These graphs compare the pre-cue-based feeding cohort (N = 272) with patients in the post-cue-based feeding cohort separated by years 2017 (N = 115), 2018 (N = 103), and 2019 (N = 96). The values of PMA are in days. Medians are shown, and the error bars are the interquartile ranges.

Feeding outcomes of the pre-protocol cohort were compared to each year in the post-protocol cohort (Fig. 3). For median PMA in days at first PO, a statistically significant decrease was noted from 240 (IQR: 235–248) in the pre-cohort to 239 (IQR: 233–244) in 2017, 237 (IQR: 232–241) in 2018, and 238.5 (IQR: 234–245.8) in 2019. Similarly, a statistically significant decrease was noted in median PMA in days at full PO feeds, 250 (IQR: 243–257) in pre-cohort, 248 (IQR: 242–256) in 2017, 245 (IQR: 239–254) in 2018, and 249.5 (IQR: 241–260) in 2019. For the median length of stay in days, there was no statistically significant difference noted when the pre-cohort 55 (IQR: 38–77.75) was compared post-cohort years, 50 (IQR: 33–66) in 2017, 45 (IQR: 33–64) in 2018, and 48 (IQR: 33.25–70.5) in 2019.

## Discussion

Our project showed a decrease in the time to first PO, time to full PO, and length of stay in VLBW infants in the NICU after the implementation of a cue-based feeding protocol. The findings of our study are in line with current evidence involving moderately preterm infants, in which every week earlier that oral feeding was initiated, there was an associated decrease in the PMA of independent oral feeding achievement by 5 days^17^. In a randomized controlled trial involving 29 infants (< 30 weeks GA), early introduction of oral feeding using a structured protocol for feeding progression improved the transition time from gavage to full PO feeds^18^. The decreased length of stay as observed in our study was similar to other studies that involved infants > 28 weeks and infants < 32 weeks^15,19^.

Cue-based feeding has shown to have positive effects on short-term health outcomes in infants, demonstrating greater weight gain, fewer oxygen desaturations, and decreased frequency of gavage feeding events^20^. Several quality improvement (QI) initiatives have been implemented in NICUs with emphasis on cue-based feeding, and a systematic review of the QI initiatives concluded that weight gain, time to full oral feedings, and hospital length of stay may be improved^21^. This approach also allows for early identification of infants at risk for a delay in the achievement of feeding independence in order to implement tailored and developmentally appropriate interventions to succeed in the PO feeding^22^.

This protocol has implications not just for providers in the NICU, as parents should be encouraged to participate in feeding when present. Feeding cues and techniques should be reinforced by bedside caregivers, and implementation of cue-based feeding can promote parental involvement in the feeding process for preterm infants^23^. Increased parental involvement will improve comfort and confidence of parents in caring for a premature infant in the NICU.

The cue-based protocol in our unit assesses both breastfeeding and bottle-feeding readiness. Breastfed infants were subsequently assessed with a breastfeeding quality score. Though there is a large emphasis on the benefits of breast milk in preterm infants, feeding at the breast is often overlooked in the NICU. First oral feed at the breast and breastfeeding subsequently have been shown to increase the duration of breastmilk feeding^24^. It will also help in preparing mothers for breastfeeding after discharge^25^.

Comorbidities in premature infants such as bronchopulmonary dysplasia (BPD), can prolong feeding readiness and initiation. based on their respiratory support requirements. There is inter-unit and inter-provider variation in starting PO in infants with BPD and on respiratory support. This is perceived as a barrier to the initiation and continuation of feeding protocols, along with the lower nurse-to-infant ratio. Our unit has established guidelines for infants on respiratory support for initiation of PO feeding. These guidelines have remained the same, along with the nurse-to-patient ratio, throughout both pre- and post-implementation.

Adverse events during feeding (desaturations below 85%, apnea > 20 s, and bradycardia) and unit-specific protocols on caffeine discontinuation can prolong the length of stay in preterm infants who can take all PO feeds. A decrease in the adverse events with cue-based feeding has been reported^12,26^. These events leading to a delayed hospital discharge can increase healthcare costs both for hospitals and patient families. Interventions in the NICU that can decrease the length of stay by 1 week can translate to average cost savings of $10,500 using an average hospital-day cost of $1500^27^.

The results of the study encouraged the team to continue using the cue-based feeding protocol. Though encouraging results were observed in the first 2 years post-implementation, they were not sustained in the third year. This was concerning, and several factors, including lack of awareness and experience level of new caregivers, may have contributed. Increasing awareness of cue-based feeding effectiveness and implementing a structured continued education program regarding its use, implementing ongoing audits, and inclusion of parents may help sustain the improvement.

Timely identification of readiness to PO is important. Cue-based feeding protocols have shown to be helpful, along with skilled caregivers, to assist infants in creating a pleasurable feeding experience, minimizing stress, and maximizing intake^28^. This study adds to our understanding of the effectiveness of cue-based feeding protocols, particularly in VLBW infants. Our study, along with the existing evidence, will help make cue-based feeding a standard practice in the NICUs. This cue-based approach will help in process optimization and minimize variability, which will eventually help preterm infants attain feeding milestones appropriately and in a timely fashion.

The study is limited by being a single-center retrospective study, being unable to control for the level of training/experience of the caregivers when using the protocol. Changes in unit policy or processes that may have occurred between two cohorts might have impacted the feeding readiness and length of stay in NICU. Also, we were unable to analyze the outcomes separately in infants who were fed directly at the breast and by the bottle.

## Conclusions

Cue-based feeding is associated with decreasing the time to first PO, time to full PO, and length of NICU stay. This enabled the infants to be discharged earlier, thus decreasing the financial burden on both patient families and the health care system. Sustaining these positive results requires time, education, ownership, and an interdisciplinary approach.

## Data Availability

The datasets generating during and/or analyzed during the current study are available from the corresponding author on reasonable request.
